# Associations of sex-related and thyroid-related hormones with risk of metabolic dysfunction-associated fatty liver disease in T2DM patients

**DOI:** 10.1186/s12902-024-01618-0

**Published:** 2024-06-07

**Authors:** Weihong Lu, Shangjian Li, Yuhua Li, Jingqi Zhou, Kai Wang, Ning Chen, Zhibin Li

**Affiliations:** 1 Department of Gynecology, Zhongshan Hospital (Xiamen), Fudan University, Xiamen, China; 2 Xiamen Clinical Research Center for Cancer Therapy, Xiamen, China; 3 Department of Endocrinology, Zhongshan Hospital (Xiamen), Fudan University, Xiamen, China; 4 School of Mechanics and Civil Engineering, China University of Mining & Technology- Beijing, Beijing, China; 5 Epidemiology Research Unit Translational Medicine Research Center, School of Medicine, The First Affiliated Hospital of Xiamen University, Xiamen University, No.55 Zhenhai Road, Xiamen, 361003 China

**Keywords:** Sex hormone, Follicle-stimulating hormone, Thyroid hormone, MAFLD, Diabetes

## Abstract

**Background:**

We aimed to examine sex-specific associations between sex- and thyroid-related hormones and the risk of metabolic dysfunction-associated fatty liver disease (MAFLD) in patients with type 2 diabetes mellitus (T2DM).

**Methods:**

Cross-sectional analyses of baseline information from an ongoing cohort of 432 T2DM patients (185 women and 247 men) in Xiamen, China were conducted. Plasma sex-related hormones, including estradiol (E2), follicle-stimulating hormone (FSH), luteinizing hormone (LH), prolactin (PRL), progesterone, and total testosterone (TT), and thyroid-related hormones, including free triiodothyronine (FT3), free thyroxine (FT4), thyroid-stimulating hormone (TSH), and parathyroid hormone (PTH), were measured using chemiluminescent immunoassays. MAFLD was defined as the presence of hepatic steatosis (diagnosed by either hepatic ultrasonography scanning or fatty liver index (FLI) score > 60) since all subjects had T2DM in the present study.

**Results:**

Prevalence of MAFLD was 65.6% in men and 61.1% in women with T2DM (*P* = 0.335). For men, those with MAFLD showed significantly decreased levels of FSH (median (interquartile range (IQR)):7.2 (4.9–11.1) vs. 9.8 (7.1–12.4) mIU/ml) and TT (13.2 (10.4–16.5) vs. 16.7 (12.8–21.6) nmol/L) as well as increased level of FT3 (mean ± standard deviation (SD):4.63 ± 0.68 vs. 4.39 ± 0.85 pmol/L) than those without MAFLD (all p-values < 0.05). After adjusting for potential confounding factors, FSH and LH were negative, while progesterone was positively associated with the risk of MAFLD in men, and the adjusted odds ratios (ORs) (95% confidence intervals (CIs)) were 0.919 (0.856–0.986), 0.888 (0.802–0.983), and 8.069 (2.019–32.258) (all p-values < 0.05), respectively. In women, there was no statistically significant association between sex- or thyroid-related hormones and the risk of MAFLD.

**Conclusion:**

FSH and LH levels were negative, whereas progesterone was positively associated with the risk of MAFLD in men with T2DM. Screening for MAFLD and monitoring sex-related hormones are important for T2DM patients, especially in men.

## Background

Type 2 diabetes mellitus (T2DM) accounts for 90% of diabetes cases, of which the global prevalence is estimated to increase to 12.2% (783.2 million) for those with 20–79 years old in 2045. The related health expenditure is projected to reach 1,054 billion USD worldwide, and therefore, has a heavy global public health and economic burden [1]. Metabolic dysfunction-associated fatty liver disease (MAFLD), formerly known as non-alcoholic fatty liver disease (NAFLD), is now defined in patients with both hepatic steatosis and any of the following three metabolic conditions: overweight/obesity, diabetes mellitus, or evidence of metabolic dysfunction in lean individuals. It affects 25% of the global population and has become another common chronic non-communicable disease [2, 3]. T2DM and MAFLD share some common pathophysiological mechanisms, such as insulin resistance; therefore, one could lead to another or vice versa [4].

Sex hormones are known to play key roles in body fat metabolism and distribution and are therefore associated with metabolic diseases, such as metabolic syndrome, T2DM, and cardiovascular disease [5, 6]. Previous studies have found that some sex-related hormones are associated with MAFLD/NAFLD; however, the results have been inconsistent and less well explored. Based on a cross-sectional analysis of 732 middle-aged and elderly community-dwelling residents, Cao et al. found that sex hormone-binding globulin (SHBG) and follicle-stimulating hormone (FSH) were negatively associated with MAFLD and liver fat content (LFC) in women, and that SHBG was negatively correlated with MAFLD and LFC in men [7]. However, they failed to find that other sex-related hormones such as estradiol (E2), total testosterone (TT), and luteinizing hormone (LH) were significantly associated with either MAFLD or LFC. In the Rotterdam Study of 755 men and 1109 women, lower levels of SHBG were found to be significantly associated with NAFLD in both sexes, while lower testosterone was associated with NAFLD only among women [8]. The same authors further conducted a meta-analysis of 16 studies and found that lower SHBG levels were associated with NAFLD in both sexes, whereas testosterone was associated with NAFLD only in men, but not in women [8].

Previous studies have shown that thyroid hormones play important roles in the regulation of metabolism, thermogenesis, and food intake, and that thyroid dysfunction leads to obesity, insulin resistance, and related metabolic complications such as metabolic syndrome [9]. As for the relationship between different thyroid hormones and MAFLD/NAFLD, The Rotterdam Study found that higher free thyroxine (FT4) was associated with decreased risk of NAFLD and hypothyroidism increased the risk of NAFLD [10]. However, there are still controversial results that free triiodothyronine (FT3) is positively associated with the risk of NAFLD and NAFLD progression in euthyroid women [11], and that thyroid-stimulating hormone (TSH) levels are significantly positively correlated with the degree of liver steatosis, but neither FT3 nor FT4 are related to NAFLD [12].

It should be noted that the inconsistent results between sex-related and thyroid-related hormones and MAFLD/NAFLD were further complicated by the different methods used for diagnoses of MAFLD/NAFLD, as well as different participants of various ages [7–13]. Therefore, sex-specific associations between sex-related and thyroid-related hormones and MAFLD remain unclear and need to be explored further, especially in T2DM patients. In the present study with 432 T2DM patients, we first aimed to determine the independent associations between different components of sex-related and thyroid-related hormones and the risk of MAFLD in all T2DM patients. Second, we aimed to explore the sex-specific associations of these sex- and thyroid-related hormones with the risk of MAFLD in men and women with T2DM separately.

## Methods

### Study design

Patient selection, diagnosis T2DM and clinical measurements have been previously described [14, 15]. Briefly, 490 T2DM patients hospitalized in the Department of Endocrinology, Zhongshan Hospital (Xiamen), Fudan University (Xiamen, China) between January 2018 and April 2020 were recruited into the present ongoing T2DM cohort. Patients were diagnosed as diabetes based on American Diabetes Association (ADA) 2018 criteria. All subjects were in-patients and hospitalized due to diabetes or diabetes-related complications. There was no acute illness and 19 of them (4.4%) having been identified as having cancers. All women subjects in the present study were postmenopausal, and none was identified as PCOS. Of these, 58 patients without complete data on sex- or thyroid-related hormones or clinical measurements were excluded, and 432 T2DM patients (247 men and 185 women) were included in the present analysis. The inclusion criteria were: (1) T2DM, (2) age ≥ 18 years, and (3) sex- and thyroid-related hormone testing and hepatic ultrasonography scanning measurements. The exclusion criteria included other types of diabetes (type 1 diabetes mellitus, secondary diabetes), severe liver and renal dysfunction, receiving or currently receiving estrogen and progesterone drugs, glucocorticoids, calcium tablets, menopause by surgical intervention or at an unnatural age, or unwillingness to participate in the study. This study was approved by the Human Research Ethics Committee of Zhongshan Hospital (Xiamen, China), Fudan University (No. B2019-015). All the participants provided written informed consent.

### Measurements

Face-to-face interviews were conducted with each patient to collect sociodemographic data, lifestyle habits, present and previous history of health, and medications. Body weight, height, BMI, waist circumference (WC), and arterial blood pressure (BP) were measured as previously described [14, 15]. Venous blood samples were collected in the morning after a 12-h overnight fast to measure fasting plasma glucose (FPG), glycosylated hemoglobin A1c (HbA1c), liver function, and lipid profiles and were tested in the clinical laboratory of Zhongshan Hospital (Xiamen), Fudan University (Xiamen, China). Serum FPG, fasting insulin, uric acid (UA), triglyceride (TG), total cholesterol (TC), high-density lipoprotein cholesterol (HDL-c), low-density lipoprotein cholesterol (LDL-c), aspartate aminotransferase (AST), alanine aminotransferase (ALT), and gamma-glutamyl transpeptidase (GGT) levels were determined using an analyzer (Roche Elecsys Insulin Test, Roche Diagnostics), as previously described [14–16]. Homeostatic model assessment of insulin resistance (HOMA-IR) was calculated using the formula: fasting serum insulin (mU/L) *fasting plasma glucose (mmol/L) /22.5 and was used to estimating insulin resistance [17]. Glycated hemoglobin (HbA1c) levels were determined using high-performance liquid chromatography (VARIANT II TURBO; Bio-Rad).

### Sex-related and thyroid-related hormones measurements

Sex-related hormones, such as estradiol (E2), luteinizing hormone (LH), follicle-stimulating hormone (FSH), prolactin (PRL), progesterone, and total testosterone (TT), as well as thyroid-related hormones, including free triiodothyronine (FT3), free thyroxine (FT4), thyroid-stimulating hormone (TSH), and parathyroid hormone (PTH), were measured using chemiluminescent immunoassay analysis (Cobas e602, Roche Diagnostics). Assay sensitivities were 0.025ng/mL for testosterone, 0.100 mIU/mL for LH, and 0.100 mIU/mL for FSH. The intra- and inter-assay coefficients of variation were < 8% and 10% for T, < 3% and 2.9% for LH, < 2.9% and 2.7% for FSH, respectively [14–16].

### Hepatic ultrasonography and definition of metabolic associated fatty liver disease (MAFLD)

Hepatic ultrasonography scanning was performed by an experienced radiologist using a GE LOGIQ P5 scanner (GE Healthcare, Milwaukee, USA) with a 4-MHz probe, and hepatic steatosis was diagnosed on the basis of characteristic sonographic features, including hepatorenal echo contrast, liver parenchymal brightness, deep beam attenuation, and vessel blurring [18]. The fatty liver index (FLI) score was calculated using the formula FLI = e^y^/(1 + e^y^) × 100, where y = 0.953 × ln (triglycerides, mg/dl) + 0.139 × BMI (kg/m^2^) + 0.718 × ln(GGT, U/L) + 0.053 × waist circumference (cm) – 15.745 [19]. A cutoff FLI score of > 60 was used to define hepatic steatosis in addition to the hepatic ultrasonography diagnosis [20]. Fibrosis-4 (FIB-4) score was calculated for each subject based on the formula: FIB-4 = age ([y] × AST [U/L]) / ((PLT [10^9^/L]) × (ALT [U/L])^1/2^), and a cutoff FIB-4 score > 3.25 was used to define advanced hepatic fibrosis [21], which was treated as one of the exclusion criteria in the present study. Fatty liver was diagnosed by either hepatic ultrasonography diagnosis of hepatic steatosis or FLI score > 60. Since all participants in the present study had T2DM, patients diagnosed with fatty liver were defined as MAFLD based on the current international consensus definition for MAFLD [2, 3, 22].

### Statistical analyses

Data are presented as mean ± standard deviation (SD) for continuous variables, which followed the approximation of normal distributions; median (interquartile range (IQR)) for non-normally distributed continuous variables; or number and percentage for categorical variables. Differences between subjects with MAFLD (yes vs. no) were analyzed using one-way analysis of variance (ANOVA) for normally distributed continuous variables, the Wilcoxon rank-sum test for non-normally distributed continuous variables, and the chi-square test for categorical variables. Multivariable logistic regression analyses were used to calculate the adjusted odds ratios (ORs) and 95% confidence intervals (CIs) of per SD increase in sex-related and thyroid-related hormones with the risk of MAFLD in all patients as well as in men and women separately, with adjustment for potential confounders, including sex, age, smoking and drinking habits, BMI, diabetes duration, HbA1c, diabetes medical treatment, triglycerides, and HDL-C. Co-variables chosen as potential confounding variables in the multivariable regression analyses were based on their significant associations with both MAFLD and sex-related and thyroid-related hormone levels. Furthermore, some traditionally and clinically relevant confounding variables were also included, even though their associations with MAFLD were not statistically significant. Since BMI and TG were incorporated in the algorithm of FLI, these two potential confounding variables were also excluded in the further multivariable regression analyses, but results on the associations of sex-related and thyroid-related hormones with risk of MAFLD did not change much (data not shown). Furthermore, since inflammation can alter many of the hormones and that should be adjusted for in the multivariable regression analyses, we tried to include C-reactive protein (CRP), an index of inflammation, as another co-variable in the multivariable regression analyses, but the results did not change (data not shown).

All p-values were two-sided, and statistical significance was set at *p* < 0.05. All statistical analyses were performed using Stata14.0 (StatCorp, College Station, TX, USA).

## Results

### Demographic and clinical characteristics stratified across MAFLD in all patients

For all the 432 T2DM patients, the mean (± SD) of age was 55.8 ± 12.5 years and median (IQR) of duration of T2DM was 6.0 (1.0–10.0) years. For all the 432 T2DM patients, 115 and 252 were diagnosed as hepatic steatosis by using FLI > 60 and by ultrasound separately; and finally, a total of 275 patients were diagnosed as MAFLD. Among them, 275 (63.7%) were identified as MAFLD and had a significantly higher FLI than controls. Table 1 shows the differences in demographic and clinical characteristics stratified by MAFLD (yes vs. no) for all patients. Generally, patients with MAFLD, compared to those without, showed significantly higher levels of body weight, BMI, waist circumference, diastolic BP, HOMA-IR, TG, HDL-c, blood uric acid, CRP, AST, ALT, and GGT, and were more likely to drink regularly (all p-values < 0.05). Regarding sex-related hormones, patients with MAFLD showed significantly lower levels of FSH only (median (IQR):11.1 (6.2–38.8) vs. 13.1 (7.6–44.8), *p* = 0.017), but not others such as E2, LH, PRL, progesterone, or TT, compared with those without MAFLD. FT3 (4.48 ± 0.83 pmol/L vs. 4.23 ± 0.76 pmol/L, *p* = 0.003) but no other thyroid-related hormones, such as FT4, TSH or PTH, was significantly increased in those with MAFLD than those without.

**Table 1:** Demographic, clinical characteristics, sex-related and thyroid-related hormones of subjects stratified by MAFLD in T2DM patients

	MAFLD		All	
	No	Yes		P value
**Demographics and clinical characteristics**				
N (%)	157 (36.3)	275 (63.7)	432 (100.0)	
Woman Sex (n, %)	72 (45.9)	113 (41.1)	185 (42.8)	0.335
Age (years)	57.1±10.9	55.0±13.3	55.8±12.5	0.092
Ever smoking (n (%))	54 (34.4)	95 (34.6)	149 (34.5)	0.988
Ever drinking (n (%))	27 (17.2)	71 (25.8)	98 (22.7)	0.040*
Weight (kg)	60.0±9.5	72.4±11.9	67.9±12.6	<0.001*
BMI (kg/m^2^)	22.5±2.5	26.6±3.8	25.1±3.9	<0.001*
Waist (cm)	81.5±8.0	93.1±10.1	88.4±10.9	<0.001*
Systolic blood pressure (mmHg)	129.4±17.1	132.2±16.5	131.2±16.8	0.086
Diastolic blood pressure (mmHg)	80.5±9.5	83.8±10.8	82.6±10.5	0.002*
Diabetes duration (years)	8.0 (1.0-11.0)	5.0 (1.0-10.0)	6.0 (1.0-10.0)	0.265
Fasting plasma glucose (mmol/L)	8.27±3.56	8.98±3.77	2.15±0.86	0.060
HbA1c (%)	9.44±2.54	9.25±2.01	9.32±2.21	0.397
HOMA-IR (*10^-6^mol*IU*L^-2^)	1.54 (1.03-2.53)	3.35 (2.15-4.92)	2.61(1.54-4.53)	<0.001*
Triglyceride (mmol/L)	1.17 (0.91-1.55)	2.00 (1.46-2.92)	1.63 (1.18-2.46)	<0.001*
Total cholesterol (mmol/L)	4.51±1.10	4.67±1.18	4.61±1.15	0.167
HDL-cholesterol (mmol/L)	1.24±0.36	1.01±0.30	1.09±0.34	<0.001*
LDL-cholesterol (mmol/L)	2.66±1.03	2.57±0.99	2.60±1.01	0.339
Blood uric acid (mmol/L)	312.1±82.8	376.7±105.6	353.2±102.6	<0.001*
AST (U/L)	16 (13-20)	18 (15-24)	17 (14-23)	<0.001*
ALT (U/L)	17 (13-22.5)	23 (16-34)	20 (14-29)	<0.001*
GGT (U/L)	19 (15-27)	33 (23-48)	27 (18.5-42)	<0.001*
C-reactive protein (mg/L)	0.8 (0.5-1.7)	1.6 (0.8-3.3)	1.3 (0.7-2.8)	<0.001*
Diabetes treatment (n (%))				
Biguanides	70 (44.6)	143 (52.0)	213 (49.3)	0.129
Glycosidase inhibitor	41 (26.1)	51 (18.5)	92 (21.3)	0.067
Sulfonylureas	52 (33.1)	82 (29.8)	134 (31.0)	0.491
TZD	7 (4.5)	26 (9.5)	33 (7.6)	0.059
Glinides	11 (7.0)	20 (7.3)	31 (7.2)	0.393
GLP-1 agonists	0 (0.0)	1 (0.4)	1 (0.2)	0.449
DPP-4 inhibitors	29 (18.5)	40 (14.5)	69 (16.0)	0.291
SGLT-2 inhibitors	1 (0.6)	1 (0.4)	2 (0.5)	0.689
Any oral hypoglycemic medications use	109 (69.4)	196 (71.3)	305 (70.6)	0.685
Insulin use	57 (36.3)	73 (26.6)	130 (30.1)	0.033*
**Sex-related hormones**				
Estradiol (E2, pmol/L)	67.5 (19.6-121.9)	82.1 (29.2-119)	77.8 (25.1-11.7)	0.633
Luteinizing hormone (LH, mIU/ml)	11 (6.6-25.8)	9.5 (6.6-19.6)	10 (6.6-22.1)	0.097
Follicle-stimulating hormone (FSH, mIU/ml)	13.1 (7.6-44.8)	11.1 (6.2-38.8)	11.9 (6.6-41.6)	0.017*
Prolactin (PRL, mIU/L)	319.5 (236.1-455)	307.7 (233.7-429.3)	311.3 (233.8-438.9)	0.688
Progesterone (nmol/L)	0.40 (0.16-0.70)	0.40 (0.20-0.70)	0.40 (0.20-0.70)	0.219
Total testosterone (TT, nmol/L)	8.4 (0.4-17.8)	9.3 (0.6-14.2)	8.95 (0.5-14.7)	0.470
**Thyroid-related hormones**				
Free triiodothyronine (FT3, pmol/L)	4.23±0.76	4.48±0.83	4.39±0.81	0.003*
Free thyroxine (FT4, pmol/L)	16.7±2.4	17.0±2.7	16.9±2.7	0.334
Thyroid stimulating hormone (TSH, mIU/L)	1.77 (1.22-2.78)	1.91 (1.27-2.88)	1.87 (1.26-2.87)	0.350
Parathyroid hormone (PTH, ng/L)	34.1±18.7	36.4±15.2	35.5±16.6	0.177
**MAFLD indices**				
FLI	20.8±15.4	57.5±22.7	42.6±26.9	<0.001*
FIB-4 score	1.19±0.86	1.09±0.64	1.13±0.73	0.199

* *p*<0.05. Abbreviations: ALT, alanine transaminase; AST, aspartate transaminas; BMI, body mass index; DPP-4, dipeptidyl peptidase 4; FIB-4, Fibrosis-4; FLI, fatty liver index; GLP-1, glucagon-like peptide 1; GGT, gamma-glutamyl transpeptidase; HDL, high-density lipoprotein; HOMA-IR, homeostasis model assessment - insulin resistance; LDL, low-density lipoprotein cholesterol; MAFLD, metabolic associated fatty liver disease; SGLT-2, Sodium glucose co-transporter 2; T2DM, type 2 diabetes mellitus; TZD, trazodone

### Demographic and clinical characteristics stratified across MAFLD in men and women

Among the 432 T2DM patients, 185 were women and 247 were men with the means (± SDs) of ages of 59.7 (± 10.7) and 52.8 (± 12.9) years, respectively (*p* < 0.001). Of these, 113 (61.1%) women and 162 (65.6%) men were identified to have MAFLD. Table 2 shows the differences in the demographic and clinical characteristics stratified by MAFLD (yes vs. no) in men and women. For both women and men, similar to all patients with T2DM, patients with MAFLD showed significantly higher body weight, BMI, waist circumference, HOMA-IR, TG, HDL-c, blood uric acid, CRP, AST, ALT, GGT, and FLI than those without MAFLD. As for sex- and thyroid-related hormones, men with MAFLD showed significantly lower FSH and TT levels as well as higher FT3 levels than those without MAFLD. However, in women with T2DM, there was no significant difference in either sex- or thyroid-related hormone levels between those with MAFLD compared with those without MAFLD.

**Table 2:** Demographic, clinical characteristics, sex-related and thyroid-related hormones of subjects stratified by MAFLD in men and women T2DM patients

	Women (*n*=185)			Men (*n*=247)		
	Non-MAFLD	MAFLD	P value	Non-MAFLD	MAFLD	P value
**Demographics and clinical characteristics**						
N (%)	72 (38.9)	113 (61.1)		85 (34.3)	162 (65.6)	
Age (years)	58.7±10.1	60.3±11.0	0.311	55.8±11.5	51.3±13.5	0.010*
Ever smoking (n (%))	3 (4.2)	3 (2.7)	0.559	51 (60.0)	92 (56.8)	0.627
Ever drinking (n (%))	2 (2.8)	1 (0.9)	0.320	25 (29.4)	70 (43.2)	0.034*
Weight (kg)	54.3±7.9	65.1±9.6	<0.001*	64.9±8.0	77.5±10.8	<0.001*
BMI (kg/m^2^)	22.3±2.8	26.5±4.4	<0.001*	22.6±2.2	26.6±3.3	<0.001*
Waist (cm)	79.0±8.5	90.0±8.1	<0.001*	83.7±6.9	95.4±10.8	<0.001*
Systolic blood pressure (mmHg)	131.9±17.7	134.8±17.9	0.274	127.2±16.3	130.4±15.2	0.127
Diastolic blood pressure (mmHg)	80.3±10.6	82.5±11.2	0.185	80.8±8.6	84.7±10.6	0.004*
Diabetes duration (years)	8.5 (2.5-12.0)	8.0 (2.0-12.0)	0.817	6.0 (1.0-10.0)	4.0 (0.9-10.0)	0.296
Fasting plasma glucose (mmol/L)	8.37±3.84	8.62±3.55	0.669	8.18±3.32	9.24±3.91	0.039*
HbA1c (%)	9.40±2.46	9.07±1.88	0.312	9.47±2.62	9.37±2.08	0.749
HOMA-IR (*10^-6^mol*IU*L^-2^)	1.56 (1.19-2.75)	3.66(2.30-6.97)	<0.001*	1.52 (1.02-2.53)	3.12(1.98-4.62)	<0.001*
Triglyceride (mmol/L)	1.27 (0.94-1.69)	1.92 (1.45-2.83)	<0.001*	1.10 (0.90-1.42)	2.11 (1.46-2.96)	<0.001*
Total cholesterol (mmol/L)	4.60±1.19	4.71±1.18	0.552	4.43±1.01	4.64±1.19	0.166
HDL-cholesterol (mmol/L)	1.34±0.41	1.08±0.30	<0.001*	1.15±0.29	0.96±0.29	<0.001*
LDL-cholesterol (mmol/L)	2.60±1.09	2.55±1.03	0.339	2.72±0.97	2.58±0.97	0.288
Blood uric acid (mmol/L)	293.4±77.2	352.5±99.8	<0.001*	327.9±84.5	393.6±106.6	<0.001*
AST (U/L)	16 (13-20)	17 (15-24)	0.010*	16.5 (14-20)	19 (15-24)	0.003*
ALT (U/L)	15.5 (11.5-21.5)	18 (14-30)	0.002*	17 (14-23)	25 (18-37)	<0.001*
GGT (U/L)	17 (14-26.5)	28 (21-39)	<0.001*	20 (15-27)	37 (26-52)	<0.001*
C-reactive protein (mg/L)	0.9 (0.5-1.5)	1.7 (0.9-3.3)	<0.001*	0.8 (0.5-1.8)	1.6 (0.7-3.3)	<0.001*
Diabetes treatment (n (%))						
Biguanides	39 (54.2)	61 (54.5)	0.968	31 (36.5)	82 (50.6)	0.034*
Glycosidase inhibitor	21 (29.2)	33 (29.5)	0.965	20 (23.5)	18 (11.1)	0.010*
Sulfonylureas	25 (34.7)	42 (37.2)	0.702	27 (31.8)	40 (24.7)	0.235
TZD	6 (8.3)	14 (12.4)	0.376	1 (1.2)	12 (7.4)	0.037*
Glinides	4 (5.6)	9 (8.0)	0.522	7 (8.2)	11 (6.8)	0.678
GLP-1 agonists	0 (0.0)	0 (0.0)	NA	0 (0.0)	1 (0.6)	0.468
DPP-4 inhibitors	22 (30.6)	21 (18.6)	0.065	7 (8.2)	19 (11.7)	0.395
SGLT-2 inhibitors	1 (1.4)	0 (0.0)	0.211	0 (0.0)	1 (0.6)	0.468
Any oral hypoglycemic medications use	55 (76.4)	92 (81.4)	0.409	54 (63.5)	104 (64.2)	0.917
Insulin use	22 (30.6)	38 (33.6)	0.663	35 (41.2)	35 (21.6)	0.001*
**Sex-related hormones**						
Estradiol (E2, pmol/L)	18.4 (18.4-44.9)	20.8 (18.4-46.0)	0.567	107.3 (72.6-133.2)	102.1 (77.8-124.4)	0.546
Luteinizing hormone (LH, mIU/ml)	26.7 (16.8-35.7)	22.6 (14.8-30.8)	0.097	8.1 (6.3-10.8)	7.6 (5.8-9.2)	0.111
Follicle-stimulating hormone (FSH, mIU/ml)	49.5 (35.7-66.4)	44.6 (29.8-55.8)	0.146	9.8 (7.1-12.4)	7.2 (4.9-11.1)	<0.001*
Prolactin (PRL, mIU/L)	346.3 (257.9-458.6)	328.8 (256.3-455.9)	0.844	308.3 (205.9-429.9)	291.2 (220.8.8-409.6)	0.905
Progesterone (nmol/L)	0.40 (0.16-0.75)	0.40 (0.20-0.70)	0.476	0.40 (0.20-0.70)	0.50 (0.30-0.70)	0.332
Total testosterone (TT, nmol/L)	0.4 (0.1-0.8)	0.5 (0.2-0.8)	0.212	16.7 (12.8-21.6)	13.2 (10.4-16.5)	<0.001*
**Thyroid-related hormones**						
Free triiodothyronine (FT3, pmol/L)	4.06±0.61	4.26±0.98	0.112	4.39±0.85	4.63±0.68	0.016*
Free thyroxine (FT4, pmol/L)	16.5±2.4	16.3±2.8	0.708	16.9±2.5	17.4±2.5	0.145
Thyroid stimulating hormone (TSH, mIU/L)	1.91 (1.40-2.98)	2.21 (1.26-2.93)	0.860	1.69 (1.01-2.49)	1.85 (1.28-2.74)	0.180
Parathyroid hormone (PTH, ng/L)	33.1±11.4	36.1±14.1	0.130	35.0±23.1	36.5±16.1	0.536
**MAFLD indices**						
FLI	19.8±16.5	52.9±22.6	<0.001*	21.7±14.5	60.9±22.3	<0.001*
FIB-4 score	1.08±0.48	1.20±0.69	0.178	1.28±1.07	1.02±0.60	0.142

* *p*<0.05. Abbreviations: ALT, alanine transaminase; AST, aspartate transaminas; BMI, body mass index; DPP-4, dipeptidyl peptidase 4; FIB-4, Fibrosis-4; FLI, fatty liver index; GLP-1, glucagon-like peptide 1; GGT, gamma-glutamyl transpeptidase; HDL, high-density lipoprotein; HOMA-IR, homeostasis model assessment - insulin resistance; LDL, low-density lipoprotein cholesterol; MAFLD, metabolic associated fatty liver disease; SGLT-2, Sodium glucose co-transporter 2; T2DM, type 2 diabetes mellitus; TZD, trazodone

### Associations between sex- and thyroid-related hormones and risk of MAFLD in all patients

Table 3 shows the unadjusted and adjusted ORs and 95% CIs per SD increase in sex-related and thyroid-related hormone levels with risks of MAFLD using logistic regression analyses for all subjects. For sex-related hormones, higher LH, FSH, and progesterone levels were significantly associated with a decreased risk of MAFLD, and the unadjusted ORs (95%CIs) for each SD increase were 0.796 (0.656–0.967), 0.804 (0.663–0.976) and 0.763 (0.598–0.975) (all *p* < 0.05), respectively. E2, PRL, and TT levels were not significantly associated with the risk of MAFLD in any of the patients. Regarding thyroid-related hormones, higher FT3, but not FT4, TSH, or PTH, was significantly associated with an increased risk of MAFLD, and the unadjusted OR (95%CI) per SD increase in FT3 was 1.452 (1.139–1.851, *p* = 0.003). After adjusting for all potential confounding factors, including sex, age, smoking and drinking habits, BMI, diabetes duration, HbA1c, diabetes medical treatment, triglycerides, and HDL-C, none of the sex-related and thyroid-related hormones were independently associated with the risk of MAFLD in all T2DM patients.

**Table 3:** Unadjusted and adjusted odds ratios (ORs) of per SD increases of sex-related and thyroid-related hormones for risk of MAFLD in all T2DM patients

	Unadjusted			Adjusted ^†^		
Sites	OR	95% CI	P value	OR	95% CI	P value
**Sex-related hormones**						
Estradiol (E2, pmol/L)	0.922	0.761–1.116	0.402	0.855	0.675–1.083	0.194
Luteinizing hormone (LH, mIU/ml)	0.796	0.656–0.967	0.022*	0.985	0.959–1.012	0.269
Follicle-stimulating hormone (FSH, mIU/ml)	0.804	0.663–0.976	0.027*	0.994	0.979–1.010	0.484
Prolactin (PRL, mIU/L)	0.959	0.793–1.160	0.669	0.930	0.755–1.145	0.493
Progesterone (nmol/L)	0.763	0.598–0.975	0.031*	0.938	0.868–1.013	0.105
Total testosterone (TT, nmol/L)	0.864	0.711–1.050	0.142	0.939	0.879–1.003	0.061
**Thyroid-related hormones**						
Free triiodothyronine (FT3, pmol/L)	1.452	1.139–1.851	0.003*	1.069	0.764–1.494	0.698
Free thyroxine (FT4, pmol/L)	1.103	0.904–1.347	0.333	0.987	0.730–1.336	0.934
Thyroid stimulating hormone (TSH, mIU/L)	1.483	0.911–2.414	0.113	1.469	0.792–2.726	0.222
Parathyroid hormone (PTH, ng/L)	1.164	0.932–1.454	0.181	1.072	0.800-1.438	0.640

* *p* < 0.05. ^†^ Multivariable logistic regression was adjusted for sex, age, smoking habit, alcohol consumption, BMI, diabetes duration, HbA1c, diabetes medical treatment, triglycerides and HDL-C

.

### Associations between sex- and thyroid-related hormones and the risk of MAFLD in men and women

Associations between sex- and thyroid-related hormones and the risk of MAFLD were further explored using multivariable logistic regression analyses in men and women separately. Table 4 shows that, for men with T2DM, with adjustment for all potential confounding factors, elevated LH and FSH levels were independently associated with decreased risks of MAFLD; the adjusted ORs (95% CIs) per SD increase in LH and FSH were 0.888 (0.802–0.983, *p* = 0.022) and 0.919 (0.856–0.986, *p* = 0.019), respectively, while increased progesterone was independently associated with a higher risk of MAFLD with an adjusted OR (95% CI) of 8.069 (2.019–32.258, *p* = 0.003). Moreover, not all thyroid-related hormones were independently associated with MAFLD risk in men with T2DM. For women with T2DM, with adjustment for all potential confounding factors, neither sex-nor thyroid-related hormones were independently associated with the risk of MAFLD.

**Table 4:** Unadjusted and adjusted odds ratios (ORs) of per SD increases of sex-related and thyroid-related hormones for risk of MAFLD in women and men T2DM patients

	Unadjusted			Adjusted ^†^				
Sites	OR	95% CI	P value	OR	95% CI	P value		
**Women** (***n* = 185)**								
**Sex-related hormones**								
Estradiol (E2, pmol/L)	0.927	0.763–1.126	0.444	0.888	0.671–1.176	0.408		
Luteinizing hormone (LH, mIU/ml)	0.799	0.615–1.037	0.092	0.998	0.971–1.027	0.910		
Follicle-stimulating hormone (FSH, mIU/ml)	0.807	0.611–1.066	0.131	0.999	0.982–1.016	0.915		
Prolactin (PRL, mIU/L)	1.070	0.795–1.440	0.656	0.981	0.690–1.394	0.915		
Progesterone (nmol/L)	0.944	0.895–0.997	0.037*	0.928	0.854–1.007	0.074		
Total testosterone (TT, nmol/L)	1.540	0.735–3.223	0.252	0.928	0.310–2.778	0.895		
**Thyroid-related hormones**								
Free triiodothyronine (FT3, pmol/L)	1.393	0.925–2.098	0.112	1.097	0.648–1.858	0.731		
Free thyroxine (FT4, pmol/L)	0.945	0.706–1.267	0.706	0.873	0.572–1.330	0.525		
Thyroid stimulating hormone (TSH, mIU/L)	1.437	0.789–2.617	0.236	2.138	0.756–6.045	0.152		
Parathyroid hormone (ng/L)	1.354	0.913–2.007	0.132	1.258	0.719–2.204	0.421		
**Men** (***n* = 247)**								
**Sex-related hormones**								
Estradiol (E2, pmol/L)	0.794	0.301–2.093	0.641	0.441	0.108–1.802	0.254		
Luteinizing hormone (LH, mIU/ml)	0.453	0.195–1.051	0.065	0.888	0.802–0.983	0.022*		
Follicle-stimulating hormone (FSH, mIU/ml)	0.286	0.105–0.783	0.015*	0.919	0.856–0.986	0.019*		
Prolactin (PRL, mIU/L)	0.785	0.520–1.184	0.248	0.830	0.529–1.303	0.418		
Progesterone (nmol/L)	1.493	0.629–3.547	0.364	8.069	2.019–32.258	0.003*		
Total testosterone (TT, nmol/L)	0.895	0.850–0.942	< 0.001*	0.939	0.874–1.010	0.091		
**Thyroid-related hormones**								
Free triiodothyronine (FT3, pmol/L)	1.468	1.069–2.016	0.018*	1.001	0.622–1.611	0.998		
Free thyroxine (FT4, pmol/L)	1.234	0.929–1.639	0.146	1.303	0.817–2.079	0.266		
Thyroid stimulating hormone (TSH, mIU/L)	1.707	0.746–3.904	0.205	1.190	0.736–1.926	0.478		
Parathyroid hormone (PTH, ng/L)	1.083	0.841–1.394	0.537	1.009	0.687–1.483	0.962		

* *p* < 0.05. ^†^ Multivariable logistic regression was adjusted for age, smoking habit, alcohol consumption, BMI, diabetes duration, HbA1c, diabetes medical treatment, triglycerides and HDL-C

## Discussion

In the present study of 432 T2DM patients, the prevalence of MAFLD was 61.1% and 65.6% in women and men, respectively. Men with MAFLD showed significantly lower FSH and total testosterone levels as well as higher FT3 levels than those without MAFLD, but there was no significant difference in either sex-related or thyroid-related hormones in women with T2DM and MAFLD compared with their controls. For men with T2DM, with adjustment for all potential confounding factors in the multivariable logistic regression analyses, elevated FSH and LH levels were independently associated with a decreased risk of MAFLD, while higher progesterone levels were independently associated with an increased risk of MAFLD. However, sex- or thyroid-related hormones were not independently associated with the risk of MAFLD in women and patients with T2DM.

In 2020, a panel of experts from 22 countries initiated a new definition of metabolic dysfunction-associated fatty liver disease (MAFLD), which is based on the presence of metabolic dysfunction (such as hypertension, type 2 diabetes, and dyslipidemia) rather than the absence of alcohol abuse or other chronic liver diseases, and proposed the replacement of NAFLD with MAFLD [2, 22]. Although the mechanisms underlying the positive association between MAFLD and T2DM are not entirely understood, it is well documented that both T2DM and MAFLD share insulin resistance and compensatory portal or systemic hyperinsulinemia as common pathophysiological mechanisms and that hepatic fat accumulation, alterations in energy metabolism, and inflammatory signals are involved in these two conditions. Therefore, it is worthwhile to explore and confirm the risk factors associated with MAFLD in T2DM patients are worthful and warranted [4, 23, 24].

Existing evidence has demonstrated that sex-related hormones are associated with the occurrence and development of NAFLD/MAFLD [7, 8, 25, 26]. Since different components of sex-related hormones were tested, and the age or ethnicity of subjects varied widely in different studies, it was not surprising that there were conflicting results on the associations between sex-related hormones and NAFLD/MAFLD. Moreover, their associations became more complicated when the definitions and diagnostic criteria for NAFLD/MAFLD were quite different among these studies. Several noninvasive imaging technologies, such as ultrasonography, computerized tomography, and magnetic resonance imaging, have been proposed as methods to diagnose fatty liver, as liver biopsy is expensive, invasive, and cannot be easily adopted, although it is still regarded as the gold standard [27]. Nowadays a few noninvasive methods which are calculated based on readily available anthropometric and biological parameters have been recommended to define NAFLD/MAFLD, such as fatty liver index (FLI) for steatosis detection and at least one of the following for liver fibrosis: non-alcoholic fatty liver disease fibrosis score (NFS), fibrosis-4 index (FIB-4) or hepamet fibrosis score (HFS) [4]. Furthermore, associations between sex-related hormones and MAFLD have not been well explored in T2DM patients.

In the present study of 247 men with T2DM, we found that those with MAFLD showed significantly lower levels of FSH and total testosterone than the controls. After adjusting for all potential confounding factors, we found that higher levels of FSH and LH were independently associated with a decreased risk of MAFLD, with adjusted ORs (95%CI) of 0.919 (0.856–0.986) and 0.888 (0.802–0.983), respectively. We also found that elevated progesterone levels were significantly associated with an increased risk of MAFLD, with an adjusted OR (95%CI) of 8.069 (2.019–32.258). But for women T2DM patients in the present study, all sex-related hormone were not significantly associated with the risk of MAFLD. Cao et al. found that FSH was negatively associated with MAFLD in women, but not in men [7]. To the best of our knowledge, this is the first study to report the significantly negative associations of FSH and LH, as well as a positive association of progesterone with the risk of MAFLD in men with T2DM. In a nationally representative sample of adults in the US, low total testosterone levels were independently associated with a higher risk of NAFLD in men [26]. Sex hormone-binding globulin (SHBG) concentration is also commonly found to be negatively correlated with NAFLD/MAFLD [7, 8, 25]. We failed to find a significant negative association between the total testosterone levels and MAFLD. Furthermore, SHBG was not tested in the present study, and serum free testosterone levels could not be calculated, which is an important limitation. The mechanisms underlying the association between FSH, LH, and progesterone levels and the risk of MAFLD in men with T2DM are not clear. In men, FSH stimulates testicular growth and enhances the production of an androgen-binding protein by the Sertoli cells, which is a component of the testicular tubule necessary for sustaining the maturing sperm cell, and LH stimulates testosterone production from the interstitial cells of the testes; therefore, FSH and LH/testosterone work in synergy and are both needed for normal spermatogenesis [28, 29]. Therefore, the present finding of the significantly negative associations of FSH and LH with the risk of MAFLD in men with T2DM was consistent with most of the existing evidence that FSH and testosterone were associated with a lower risk of NAFLD/MAFLD in both women and men [7, 8]. For women T2DM patients in the present study, we did not find any significant association between sex-related hormones (E2, LH, FSH, PRL, progesterone, or total testosterone) and the risk of MAFLD. One possible reason is that the women in our study were older and postmenopausal and had relatively lower levels of sex-related hormones, which may hamper us to find their true associations with MAFLD that could be true in the general populations.

Thyroid hormones play important roles in maintaining metabolic homeostasis, and thyroid dysfunction is associated with insulin resistance and T2DM. However, the available evidence on the association between thyroid-related hormones (FT3, FT4, or TSH) and NAFLD is inconsistent [10–12, 30, 31], and no evidence was found to explore the relationship between thyroid-related hormones and MAFLD. In the present study, although we found that T2DM patients with MAFLD showed significantly increased levels of FT3 for all and men, the association between FT3 and the risk of MAFLD became statistically non-significant after adjustment for potential confounding factors. The FT4, TSH, and PTH levels were not significantly different between men and women with MAFLD and their controls for all, men or women T2DM patients. The reasons for different findings regarding the independent relationships between thyroid-related hormones and MAFLD are not fully understood. One possible reason was that the subjects in the present study were all T2DM patients who were overweight or obese and had higher levels of insulin resistance than the general population; both conditions (obesity and insulin resistance) possibly mediated the association between thyroid dysfunction and MAFLD [30]. Therefore, further research on the association between thyroid-related hormones and MAFLD needs to be conducted, especially in different subjects.

Existing evidence on the relationship between sex-related and thyroid-related hormones and MAFLD/NAFLD was mainly conducted in the general population, while few studies have focusing on T2DM patients in China, where the prevalence of T2DM is increasing worldwide. To the best of our knowledge, this is the first study to report the independent negative associations of FSH and LH, as well as a positive association of progesterone with the risk of MAFLD in men with T2DM, although other sex-related and thyroid-related hormones were not significantly associated with MAFLD in either men or women. However, our study had several limitations. First, it was based on cross-sectional analyses of our ongoing T2DM cohort, and we could not determine the temporal sequences of the associations of sex-related and thyroid-related hormones with MAFLD. Second, the sample size was small and all 432 participants were T2DM patients sampled from one hospital in Xiamen, China; therefore, selection bias in the present study was inevitable, and we could not generalize the present findings to other populations with limited power. Third, SHBG concentration was not tested, and free testosterone could not be calculated in the present study, whereas SHBG was generally found to be significantly associated with NAFLD/MAFLD in other studies. Fouth, the female participants in the present study were older and postmenopausal, which may hamper us to find the real associations of sex-related hormone with MAFLD that could be true in the general populations. Fifth, the assay used to measure sex-related hormones is chemiluminescent immunoassay but not the gold standard assay (mass spectrometry), which may affect results on the association between sex hormones and MAFLD. Sixth, due to a few of exposure variables, risks of multi-testing and false positive results were very likely in the present analyses, especially for our relatively small sample size. Finally, healthy controls were not included in the present study, and we could not compare sex- and thyroid-related hormones between them and T2DM patients. Therefore, future studies with larger sample sizes and complete measurements of sex-related hormones, particularly those based on a prospective cohort study design, are needed. It should be noted that the present subjects were all T2DM patients from our ongoing TDM cohort and therefore our findings could only be limitedly extrapolated to those similar T2DM patients but not the general populations.

## Conclusion

MAFLD was common in T2DM patients, with a prevalence rate of approximately 63.7% in the present study. Serum FSH and LH levels were negative, whereas progesterone levels were positively associated with the risk of MAFLD in men with T2DM. However, no significant association between sex-related hormone levels and the risk of MAFLD was found in women with T2DM. Thyroid-related hormones (FT3, FT4, TSH, and PTH) were not independently associated with the risk of MAFLD in either men or women with T2DM. Therefore, our findings could be used to imply that screening for MAFLD and monitoring sex-related hormone levels are important for T2DM patients, especially in men.

## Data Availability

The data that support the findings of this study are available from the corresponding author, upon reasonable request.
